# Color tunable low cost transparent heat reflector using copper and titanium oxide for energy saving application

**DOI:** 10.1038/srep20182

**Published:** 2016-02-05

**Authors:** Goutam Kumar Dalapati, Saeid Masudy-Panah, Sing Teng Chua, Mohit Sharma, Ten It Wong, Hui Ru Tan, Dongzhi Chi

**Affiliations:** 1 Institute of Materials Research and Engineering, A*STAR (Agency for Science, Technology and Research), 2 Fusionopolis Way; Innovis, #08-03, 138634 Singapore

## Abstract

Multilayer coating structure comprising a copper (Cu) layer sandwiched between titanium dioxide (TiO_2_) were demonstrated as a transparent heat reflecting (THR) coating on glass for energy-saving window application. The main highlight is the utilization of Cu, a low-cost material, in-lieu of silver which is widely used in current commercial heat reflecting coating on glass. Color tunable transparent heat reflecting coating was realized through the design of multilayer structure and process optimization. The impact of thermal treatment on the overall performance of sputter deposited TiO_2_/Cu/TiO_2_ multilayer thin film on glass substrate is investigated in detail. Significant enhancement of transmittance in the visible range and reflectance in the infra-red (IR) region has been observed after thermal treatment of TiO_2_/Cu/TiO_2_ multilayer thin film at 500 °C due to the improvement of crystal quality of TiO_2_. Highest visible transmittance of 90% and IR reflectance of 85% at a wavelength of 1200 nm are demonstrated for the TiO_2_/Cu/TiO_2_ multilayer thin film after annealing at 500 °C. Performance of TiO_2_/Cu/TiO_2_ heat reflector coating decreases after thermal treatment at 600 °C. The wear performance of the TiO_2_/Cu/TiO_2_ multilayer structure has been evaluated through scratch hardness test. The present work shows promising characteristics of Cu-based THR coating for energy-saving building industry.

Transparent heat reflecting (THR) coating has a high reflectance at the near infrared (IR) radiation and a high transmittance at the visible region^1^,^2^. Currently, most IR reflectors are made of silver (Ag) due to its color neutrality. However, Ag is expensive. Similarly, gold (Au) exhibits optimum reflectivity spectrum of heat reflecting coating, but its potential is greatly reduced by the high price. On the other hand, copper (Cu) has high reflectivity of IR radiation and low market price compared to the Ag and Au. Towards this, we have developed the Cu-based low cost color tunable anti-scratch heat reflector coating for the THR window (THRW) application.

Conventional windows not only pass the visible solar spectrum but also allows near infrared (IR) radiation (known as the source of heat). Therefore, efficient method to save energy in buildings is to enhance the reflectance of near IR radiation without sacrificing the transmittance of the visible solar spectrum^3^,^4^,^5^. Transparent heat reflecting window (THRW) has high reflectance at the near IR radiation solar spectrum and high transmittance at the visible region. Therefore, by employing the THRW, energy saving building with reduced energy costs can be achieved by inhibiting IR radiation. The translation of production cost into low price is important to encourage the consumption of THRW for energy-saving application. However, the mass consumption of THRW is imperative to achieve this laudable outcome; thus, it is essential to develop low-cost THRW technology to attract more consumers. There are number of designs to demonstrate THR coating; among them, dielectric/metal/dielectric design is one of the most promising structures for IR reflectance and thermal isolation^6^. In this structure, thin metal film is used to reflect IR, while anti-reflection dielectric layers are employed to enhance the transmittance in visible spectrum, protect thin metal film from environmental effects and improve the growth of continuous thin metal films. Thin film of silver and gold are mainly used as the metal in dielectric/metal/dielectric structures^7^,^8^,^9^,^10^,^11^,^12^,^13^,^14^,^15^,^16^. Silver and gold show excellent performance as a heat reflector coating; however, both are expensive. On the other hand, compared with Ag, Cu is low cost metal and it is earth abundant. The production cost for Cu is also very cheap. Therefore, Cu is a suitable candidate for the development of low cost THRW. However, there are few issues need to solve before the production of Cu based THRW. Oxidation of Cu significantly affects the performance of THRW. Furthermore, the Cu based transparent heat reflector appearances brownish in color; thus it is essential to developed Cu based THR coating with neutral color. Control of inter-diffusion of elemental Cu is also another challenge. Thus, enhancing infrared (IR) reflectance and visible transmittance of solar spectrum using thin film Cu metal with neutral color is much desirable for the low cost transparent heat reflecting window application.

Metal oxides are promising candidate for solar energy harvesting and smart window applications^17^,^18^,^19^,^20^,^21^,^22^,^23^,^24^,^25^,^26^,^27^,^28^,^29^,^30^. Among them, titanium oxide (TiO_2_) is one of the most extensively investigated materials for solar energy applications^31^,^32^,^33^,^34^,^35^,^36^. High optical transmittance in the visible region, high refractive index and wide band gap of TiO_2_, make it suitable candidate for UV absorption and heat reflection coating application^32^,^33^,^34^. The strong chemical bond and mechanical stability, low production cost and availability of initial material in the earth crust make it one of the most promising candidates for THRW applications^17^,^37^,^38^. High thermodynamic stability and density also make TiO_2_ an excellent protective layer over metal layer in dielectric/metal/dielectric design. Its high absorbance of UV radiation is also especially desired in THRW application. In spite of its promising properties, only a few research articles investigated the TiO_2_/metal/TiO_2_ multilayer structure for heat reflecting coating applications^17^,^37^,^38^. High optical transmittance in the visible region is essential for the THR application. The adequate efforts have not been done to address the effect of dielectric crystallinity and metal-dielectric interface properties on the IR reflection and visible transmittance in TiO_2_/Cu/TiO_2_ structures. Furthermore, tuning of color of the Cu-based heat reflector is also not established. Therefore, it is very essential to investigate the TiO_2_/Cu/TiO_2_ multilayer for the production of low cost THRWs using sputter technique for large scale deployment of energy savings application. The sputter deposition technique has been widely accepted for industrial applications and it is also capable of batch processing^39^,^40^,^41^,^42^,^43^.

In this paper, we have investigated the sputter deposited TiO_2_/Cu/TiO_2_ multilayer thin film on glass substrate for the development of low cost and neutral color THRWs. Thin film of TiO_2_ and Cu was deposited on glass substrate at room temperature using stoichiometry TiO_2_ target and Cu target, respectively. Impact of thermal treatment of TiO_2_/Cu/TiO_2_ multilayer thin film at different temperatures was investigated in detail. Furthermore, effect of dielectric crystal quality and out diffusion of elemental oxygen and Cu on the IR reflection property of TiO_2_/Cu/TiO_2_ thin films has been discussed. By tuning the multilayer thickness and thermal treatment, the Cu-based THRWs with neutral color have been developed.

## Experimental

The TiO_2_/Cu/TiO_2_ multilayer thin film was deposited using sputter deposition technique at room temperature on borosilicate float glass substrates. Before loading borosilicate float glass substrates into sputtering chamber, all substrates were ultrasonicated in the deionized (DI) water for 10 mins and then dried with nitrogen gas flow. Pure copper and stoichiometric TiO_2_ targets were used to sputter the TiO_2_/Cu/TiO_2_ multilayer thin film on glass substrates. All depositions were performed sequentially without breaking the vacuum. The RF power was maintained at 150 W to sputter the TiO_2_ and the DC power was held at 100 W to deposit the copper metal layer. Ar gas flow rate was kept constant at 25 sccm and the deposition was done at a working pressure of 3.3 mTorr. Copper thicknesses were varying from 10 nm to 20 nm. Total thickness for TiO_2_ was varying from 20 nm to 100 nm. The TiO_2_/Cu/TiO_2_ structures were annealed at different temperatures in nitrogen ambient for 1 min with heating and cooling rate of 10 °C/sec by using rapid thermal processing (RTP) system, Jet First 150 (Jipelec).

Structural properties of the as-deposited and annealed thin films were investigated by X-ray diffraction measurements with Cu-Kα radiation and wavelength of 1.5456 Å. High resolution Philips CM300 transmission electron microscopy (HR-TEM) was carried out to study the crystallinity of TiO_2_. Double-beam Shimadzu UV-3101 UV-VIS-NIR Scanning Spectrophotometer was used to measure the optical properties. Transmittance spectra of the heat reflector were measured in the wavelength range of 300 nm to 2000 nm at normal incidence (0°). Specular reflectance attachment (angle of incidence 5°) was used to measure the relative reflectance of specular reflected light.

## Results and Discussion

**Figure 1:**
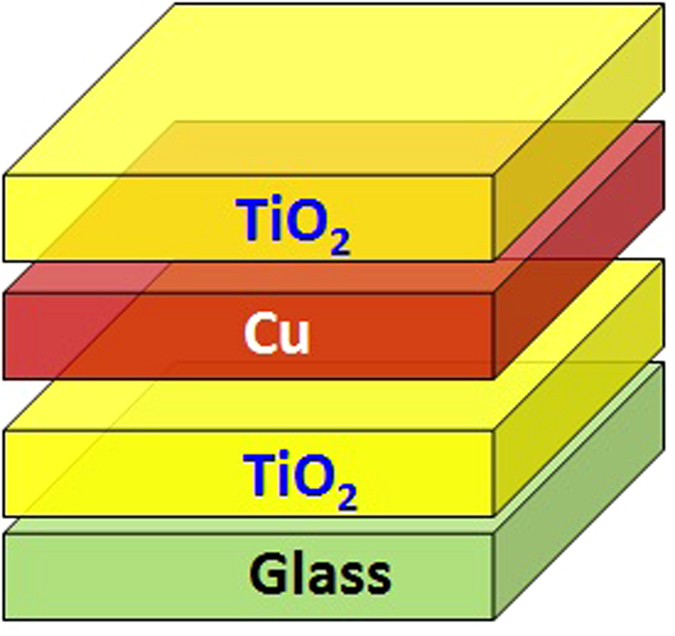
Schematic diagram of transparent heat reflector (THR) using symmetrical dielectric (TiO_2_) and identical thickness over and below Cu layer.

**Figure 2:**
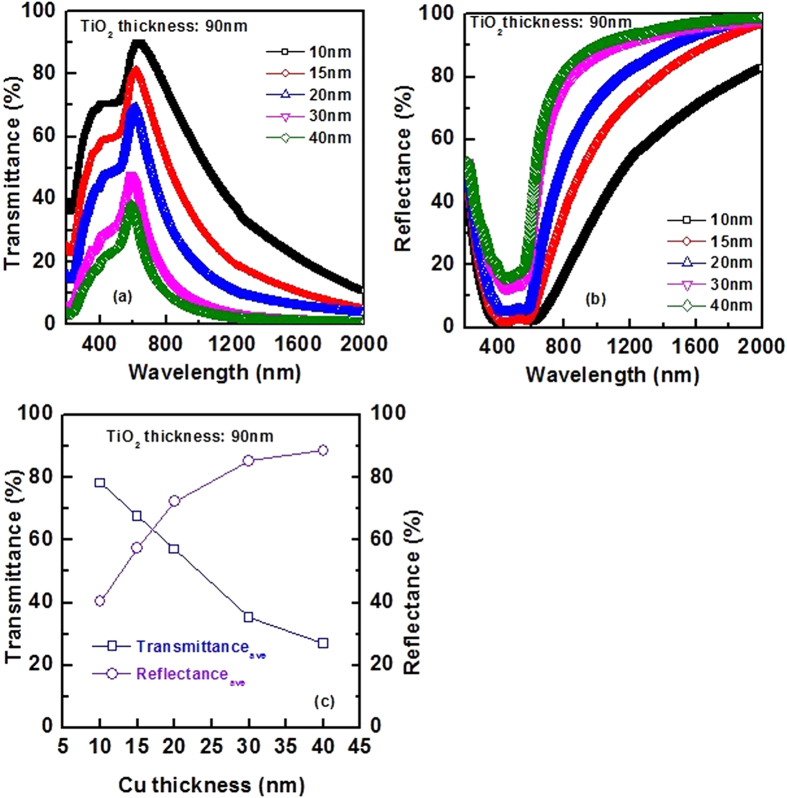
Software predicted (**a**) transmittance spectra and (**b**) reflectance spectra of TiO_2_/Cu/TiO_2_ THR with different thickness of Cu layer ranging from 10 nm to 40 nm and fixed thickness of TiO_2_. The total thickness for TiO_2_ is 90 nm. (**c**) shows average transmittance in wavelengths ranging from 400 to 700 nm and average reflectance in the wavelengths ranging from 700 to 1000 nm.

Thickness of the TiO_2_ and Cu layers plays a crucial role in the optical properties of TiO_2_/Cu/TiO_2_ transparent heat reflector (THR) coating. IMD software for modelling and analysis of multilayer films is used to evaluate the optical characteristics of TiO_2_/Cu/TiO_2_ THR and to find out suitable thickness of the TiO_2_ and Cu layers. Figure 1 shows schematic diagram of TiO_2_/Cu/TiO_2_/glass heat reflector coating. The optical transmittance and reflectance of TiO_2_/Cu/TiO_2_ THR for different thicknesses of Cu layer ranging from 10 nm to 40 nm and fixed thickness of TiO_2_ at top layer of 45 nm are presented in Fig. 2(a,b), respectively. As shown in Fig. 2(a,b), reflectance at IR wavelengths increases while the transmittance at visible wavelengths decreases by increasing the thickness of Cu layer. In the visible wavelengths, the transmittance spectra is influenced by absorption of light in the Cu thin film due to the interband electronic transitions, specially, due to the excitation of electrons from the d-band to the Fermi surface. Indeed by increasing the thickness of Cu layer there are more bound electrons available for excitation and therefore transmission reduces further. Also the bandwidth of transmittance at visible wavelengths narrows by increasing the thickness of Cu layer which is mainly originated from more free electrons of the thicker Cu layer and domination of free carrier absorption at these wavelengths. High visible transmittance and IR reflectance is the most important factor for designing the transparent heat reflector. The average of transmittance spectra at visible wavelengths ranging from 400 nm to 700 nm and average of reflectance spectra at IR wavelengths ranging from 800 nm to 2000 nm are also presented in Fig. 2(c). As shown in Fig. 2(c), the suitable thickness of Cu layer should be around 10 nm to 20 nm to get the high visible transmittance and IR reflectance for TiO_2_/Cu/TiO_2_ transparent heat reflector.

**Figure 3:**
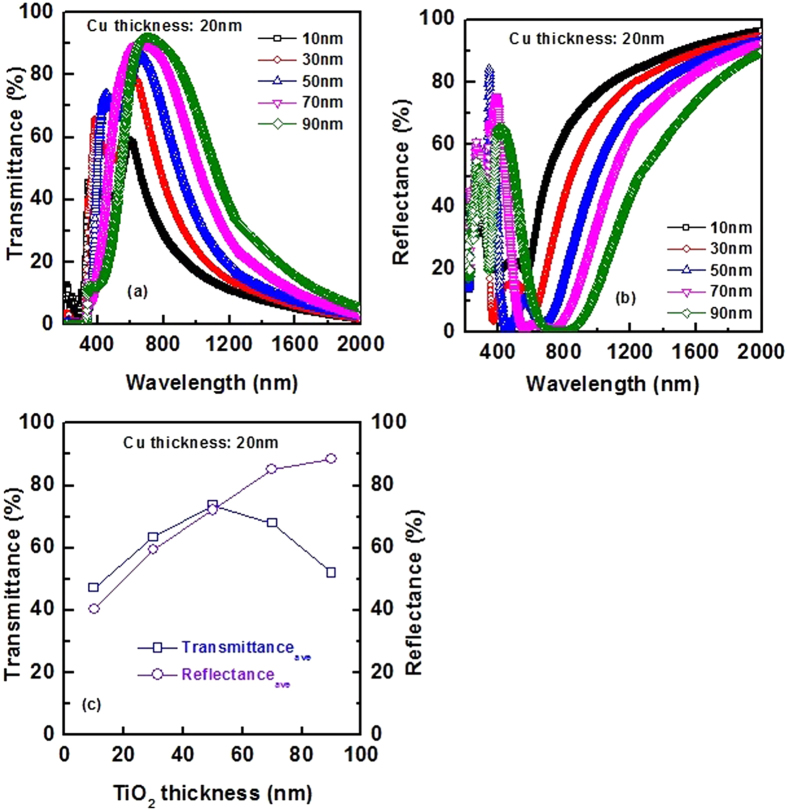
Software predicted (**a**) transmittance spectra and (**b**) reflectance spectra of TiO_2_/Cu/TiO_2_ THR with different thickness of TiO_2_ layer. The total thickness of TiO_2_ layer (top layer and bottom layer) varying from 10 to 90 nm. Thickness of Cu is 20 nm. (**c**) shows average transmittance in wavelengths ranging from 400 to 700 nm and average reflectance in the wavelengths ranging from 700 to 1000 nm.

Thin film TiO_2_ also plays a significant role on the optical spectra of TiO_2_/Cu/TiO_2_ multilayer coating. In Fig. 3, impact of TiO_2_ thickness on optical properties of TiO_2_/Cu/TiO_2_ THR coating is investigated. The thickness of Cu metal layer is fixed at 20 nm for all samples. The average of visible transmittance and IR reflectance is also presented in this figure. It is desirable to increase both visible transmittance and IR reflectance for transparent heat reflector application. However, in practice, it is challenging to increase both functions simultaneously. It is clear from Fig. 3 that the TiO_2_ with the thickness of ~50 nm shows the highest possible combination of IR reflectance and visible transmittance.

**Figure 4:**
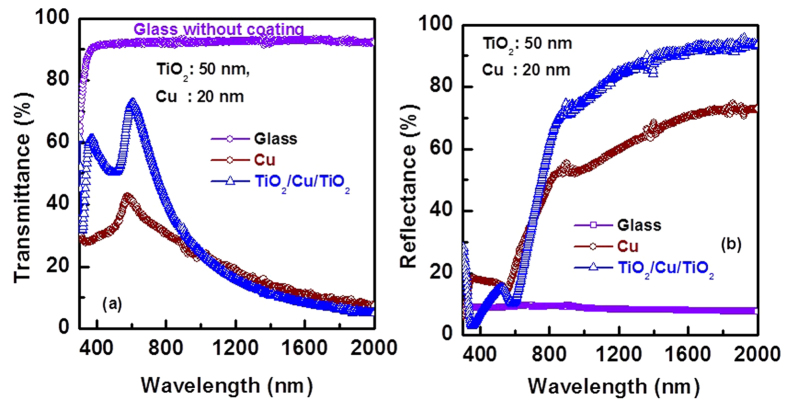
Comparison of optical (**a**) transmittance and (**b**) reflectance of glass, thin film Cu (20 nm) and TiO_2_/Cu/TiO_2_ (50 nm/20 nm/50 nm) multilayered thin films without thermal treatment.

Figure 4 shows optical transmittance and reflectance spectra for glass, thin film of Cu of thickness 20 nm, and TiO_2_/Cu/TiO_2_ multilayer coating on glass substrate. Thickness of Cu is 20 nm and total thickness of TiO_2_ (under-layer and over-layer of Cu) is 100 nm in the multilayer structure. Visible transmittance significantly improved for the TiO_2_/Cu/TiO_2_ structure compared with Cu layer (Fig. 4a). This is due to the anti-reflection property of TiO_2_ layer^27^,^28^. It is also worth to note that the IR reflection property significantly improved for TiO_2_/Cu/TiO_2_ structure, as compared with single Cu layer on glass substrate.

**Figure 5:**
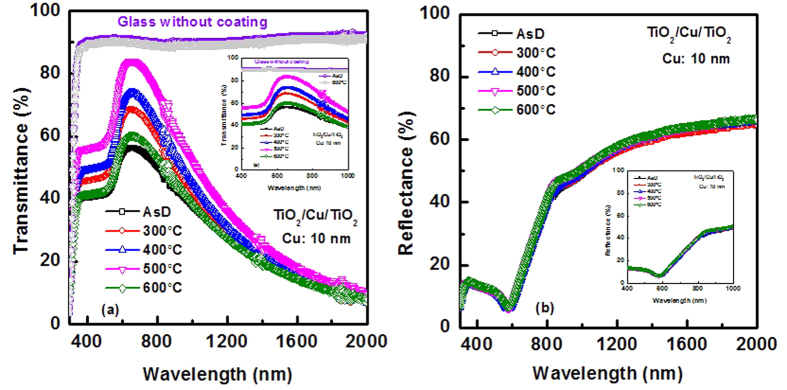
Measured (**a**) Transmittance spectra and (**b**) reflectance spectra of TiO_2_/Cu/TiO_2_ THR with and without thermal treatment. The thickness of Cu and top layer TiO_2_ is 10 nm and 25 nm, respectively. Inset of (**a,b**) show the variation of transmittance and reflectance spectra in the visible range and NIR region of the heat reflector. Transmittance spectra of plain glass with and without thermal treatment at 600 °C are also compared.

One of the most important parameters that can significantly influence the performance of metal-oxide/metal/metal-oxide THR coating and has not been completely addressed yet is the impact of metal oxide crystal quality. Rapid thermal annealing (RTA) is efficient method to improve the crystal quality and bulk oxide quality of the metal oxides^43^,^44^,^45^,^46^. In the following, effects of RTA on the sputter deposited TiO_2_/Cu/TiO_2_ multilayer coating on glass are investigated. UV-vis-NIR spectroscopy has been used to study the transmission and reflection property of the heat reflector coating. Figure 5 shows the measured transmittance and reflectance spectra of TiO_2_/Cu/TiO_2_ multilayer structure with the Cu layer thickness of 10 nm. The optical transmittance of plain glass with and without thermal treatment at 600 °C is also compared in Fig. 5(a). The optical transmittance of the plain glass is ~90% over the visible range of wavelengths irrespective of the thermal temperature. Figure 5(a) shows optical transmittance of TiO_2_/Cu/TiO_2_ multilayer coating for the as-deposited (AsD) and after thermal treatments at different temperatures. The IR reflectance spectra for the multilayer coating were shown in Fig. 5(b). For the AsD multilayer coating, visible transmittance is ~55%, while IR reflectance is ~50% at a wavelength of 1000 nm. The visible transmittance significantly improved after thermal treatment of the multilayer coating at 300 °C, 400 °C and 500 °C. The visible transmittance of ~85% is achieved, which is comparable with the glass without coating. It is worth to note that even though visible transmittance improves significantly, IR reflectance has minimal effect with thermal treatment. The IR reflectance at a wavelength of 1000 nm is ~55%. This is due to the thin metal layer. For THRW application, IR reflectance needs to be improved further to reduce the heat inside building significantly.

**Figure 6:**
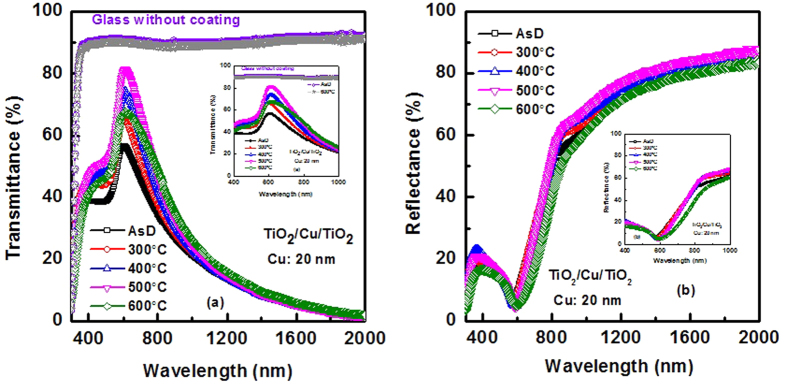
Measured (**a**) Transmittance spectra and (**b**) reflectance spectra of TiO_2_/Cu/TiO_2_ THR with Cu layer thickness of 20 nm and top TiO_2_ layer thickness of 25 nm. (**b**) Inset of (**a**,**b**) show clear variation of heat reflector properties in the visible range and NIR region. Transmittance spectra of plain glass with and without thermal treatment at 600 °C are also compared.

The heat reflection property can be improved significantly by tuning the Cu layer thickness. Figure 6(a,b) shows the transmittance and reflectance property of the multilayer structure with Cu layer thickness of 20 nm, respectively. Thickness of top TiO_2_ layer is 25 nm. The results are very promising. The visible transmittance of the multilayer coating is ~82% after thermal treatment at 500 °C and the transmittance significantly drops after wavelength of 800 nm. The IR reflectance at a wavelength of 1000 nm is 70%. Indeed, IR reflection of the multilayer coating is below 20% in the visible range and it enhanced >85% in the NIR-IR region.

**Figure 7:**
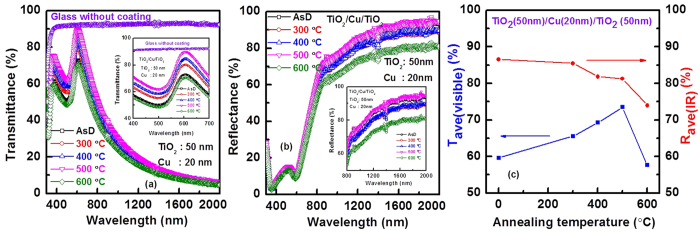
(**a**) Transmittance spectra and (**b**) reflectance spectra of TiO_2_/Cu/TiO_2_ THR with Cu layer thickness of 20 nm and top TiO_2_ layer thickness of 50 nm. (**b**) Inset of (**a,b**) show clear variation of heat reflector properties in the visible range and NIR region. (**c**) Variation of average visible transmittance and IR reflectance with annealing temperature.

**Figure 8:**
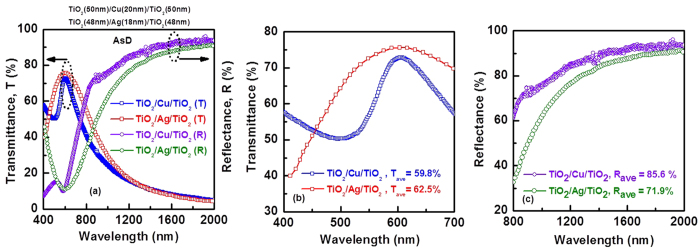
Comparison of TiO_2_/Cu/TiO_2_ performance with TiO_2_/Ag/TiO_2_. Thickness of TiO_2_ and metals are similar.

Furthermore, by increasing the thickness of TiO_2_, performance of the THR coating can be improved significantly. Visible transmittance and IR reflectance enhanced for the TiO_2_/Cu/TiO_2_ structure with 50 nm thick TiO_2_, as shown in Fig. 7a,b. The average visible transmittance (over the range of 400 nm to 700 nm) and IR reflectance (over the range of 800 nm to 2000 nm) are shown in Fig. 7c. This is the highest reported results for the Cu based heat reflecting coating^37^,^38^. Performance of the TiO_2_/Cu/TiO_2_ structure also compared with TiO_2_/Ag/TiO_2_ structure. Figure 8 shows the optical spectra for Cu-based and Ag based THR. Thicknesses of TiO_2_ and Ag are 48 nm and 18 nm, respectively. Average visible transmittance for Cu-based THR is comparable with the Ag-based heat reflector, while, average IR reflectance is slightly higher for Cu-based coating compared with the Ag-based heat reflector coating^6^.

The TiO_2_/Cu/TiO_2_ shows promising results for the transparent heat reflector coating. However, Cu-based THR coating needs thermal treatment to improve the visible transparency. For the as-deposited coating on glass substrate, the visible transparency is ~70% at a wavelength of ~600 nm. Visible transmittance improved significantly to ~90% after thermal treatment of the coating at 500 °C for 1 min. There is no significant change in IR reflection for the multilayer heat reflector coating with Cu thickness of 10 nm and 20 nm. Since the reflectance in the visible region changed minimally, the higher transmittance could be due to the improvement of TiO_2_ dielectric crystal quality. XRD and HRTEM have been employed to study the impact of thermal treatment on the heat reflector performance.

**Figure 9:**
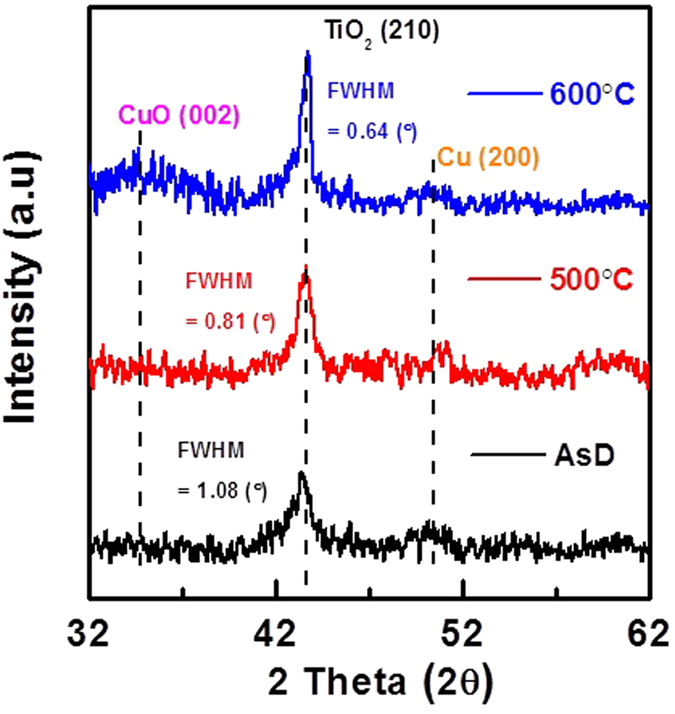
XRD spectra of the TiO_2_/Cu/TiO_2_ THR with and without thermal treatment. The crystal quality improved after annealed of the heat reflector layer. There is no sign of formation of copper oxide even after annealing at 500 °C.

Figure 9 shows the XRD spectra of TiO_2_/Cu/TiO_2_ multilayer thin film as a function of the annealing temperature. The corresponding FWHM of TiO_2_ peaks are also indicated in this figure. Rutile TiO_2_ (210) is appeared at 44.052°^47^ for the AsD and annealed samples (JCPDS# 00-021-1276). Peaks of CuO (002) and Cu (200) are observed at 35.482° and 51.5°, respectively^48^,^49^,^50^,^51^ (JCPDS# 05-0661). The intensity of Cu and CuO peaks are much lower than that of TiO_2_ (210) due to the low thickness of Cu and/or CuO layer (~10 nm) as compared to that of TiO_2_ (~40 nm). Generally, crystalline TiO_2_ exists in three different phases, namely rutile, anatase and brookite. In this structure, rutile TiO_2_ phase was observed. According to the Fig. 9, as-deposited TiO_2_ also shows rutile phase. However, intensity of XRD peak increases and corresponding FWHM reduces with the increase of annealing temperature. This indicates that the crystal quality of TiO_2_ is improved without phase change^47^,^52^. The average grain size increases with the annealing temperature, subsequently reducing the grain boundary density of the TiO_2_ film and influences the transmission of the heat reflector. The intensity of Cu peak also decreases slightly with the increase of annealing temperature, indicating partial oxidation of Cu metal thin film at high temperature. From the XRD spectra, it can also be seen that there is a presence of very thin copper oxide after annealed at 600 °C^49^,^50^,^51^. It is notable that even though, crystal quality improved after annealed at 600 °C, the heat reflecting and transparency properties are degraded due to the partial oxidation of thin metal layer and inter-diffusion of elemental Cu and oxygen.

**Figure 10:**
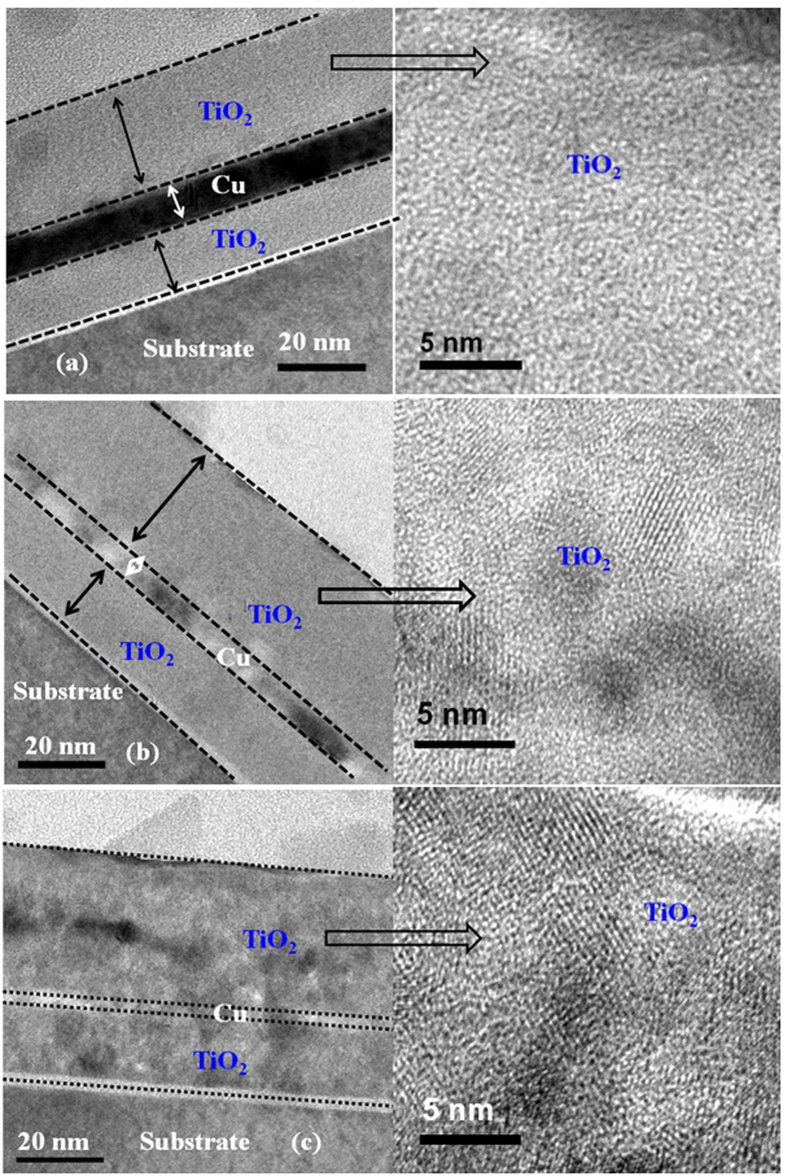
Cross-sectional high resolution TEM of the TiO_2_/Cu/TiO_2_ THR for the (**a**) as-deposited samples, after annealed (**b**) at 500 °C and (**c**) 600 °C for 1 min in nitrogen ambient. Crystal quality improved after thermal treatment; however, Cu layer partially oxidized after annealed at 600 °C.

**Figure 11:**
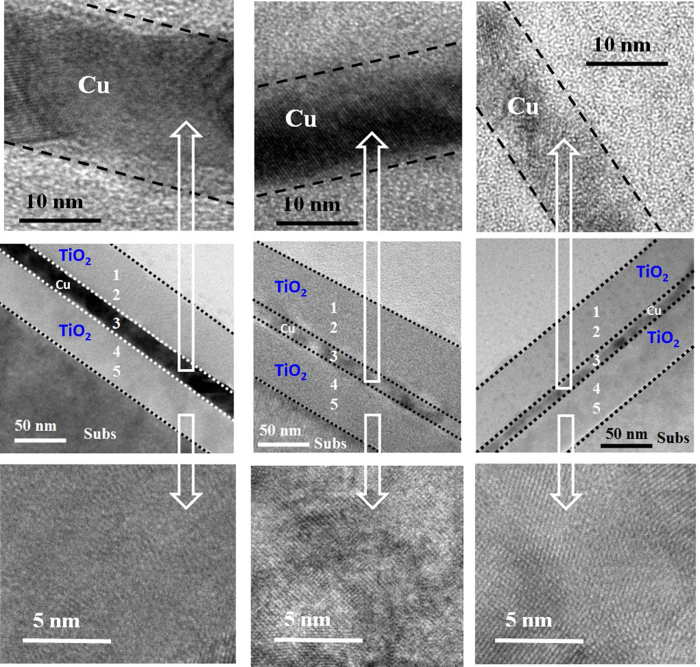
Cross-sectional high resolution TEM of the TiO_2_/Cu/TiO_2_ THR for the (**a**) as-deposited samples, after annealed at (**b**) 500 °C and (**c**) 600 °C for 1 min in nitrogen ambient for thicker TiO_2_ (total thickness ~100 nm). Crystal quality improved after thermal treatment; however, Cu layer partially oxidized after annealed at 600 °C and its thickness slightly reduces from 20 nm to 13 nm. EDX performed at different regions shown in figure through numbers.

To investigate the effects of annealing temperature on the interface properties and crystal quality of TiO_2_/Cu/TiO_2_ transparent heat reflector, high resolution TEM (HR-TEM) (Philips CM300) was employed. HR-TEM image of TiO_2_/Cu/TiO_2_ thin film for as-deposited (AsD) and annealed at 500 °C and 600 °C are presented in Fig. 10. The existence of continuous thin film of Cu of thickness ~10 nm is observed (Fig. 10(a)). For the annealed sample at 500 °C, TiO_2_ crystallinity quality has been improved as shown in Fig. 10(b). By increasing the annealing temperature up to 600 °C the crystal quality of TiO_2_ layer can be improved further, however, partial oxidation of copper metal layer and formation of copper-copper oxide interfacial layer can be observed, as shown in Fig. 10(c). The results are in agreement with the observation of XRD, where intensity of TiO_2_ peak increases with annealing temperature without phase change, and there is a presence of low intensity CuO peak at 600 °C. The thickness of metal layer is reduced from 10 nm to 8 nm after thermal treatment at 500 °C (Fig. 10(b)). For the sample annealed at 600 °C metal layer thickness is around 5 nm, indicating the partial oxidation of metal layer during thermal treatment. To investigate further about thermal impact on the TiO_2_/Cu/TiO_2_ structure, we have performed HRTEM analysis and energy dispersive X-ray analysis in TiO_2_/Cu/TiO_2_ structure with top and bottom layer TiO_2_ with thickness of 50 nm. As shown in Fig. 11, crystal quality improves significantly with the annealing temperature. It is also worth to note that for the thicker dielectric, thickness of the TiO_2_ is similar over Cu and under Cu layer. After thermal treatment at 600 °C, thickness of Cu metal layer slightly reduces to 13 nm. Symmetrical structure with identical thickness is critical to get the high visible transmittance and IR reflectance. Table 1 shows the elemental composition of Cu, Ti and O over the whole structure. From the EDX analysis, it is appeared there is negligible Cu out-diffusion into the TiO_2_. In the later section, the Cu diffusion and its impact of the performance of Cu based THR has been discussed.

**Figure 12:**
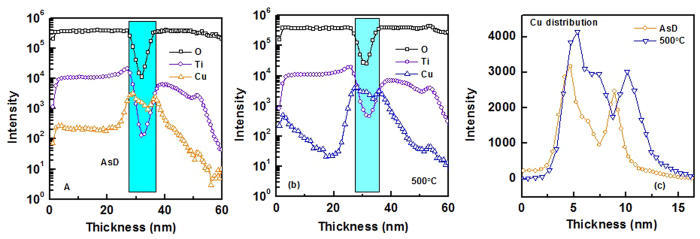
SIMS profile of Cu, Ti and O elements of the TiO_2_/Cu/TiO_2_ THR for the (**a**) as-deposited and (**b**) annealed at 500 °C.

In order to get more insight about the metal layer oxidation and inter-diffusion at metal-dielectric interface, SIMS depth profiling was used to study the elemental distribution throughout the TiO_2_/Cu/TiO_2_ multilayer coating on glass substrate. Figure 12(a,b) show SIMS depth profile of AsD TiO_2_/Cu/TiO_2_ THR and annealed sample at 500 °C, respectively. The distribution of Cu metal layer of AsD and annealed sample is also compared in Fig. 12(c). The metal-dielectric interface sharpness, which is resulted from inter-diffusion through TiO_2_ and Cu layer, is influenced by annealing temperature. Indeed, due to out-diffusion of oxygen from dielectric layer, effective thickness of copper layer is reduced and consequently the optical properties are influenced after thermal treatment at 600 °C. It is also worth noting that there is Ti hump at TiO_2_/Cu interface, which suggests formation of Ti-rich oxide at the interface.

The important aspect of transparent heat reflectors for real-life applications is the color tuning of the transparent heat reflectors and obtaining neutral color in appearance. Colors can be measured and quantified in various ways; indeed, a person’s perception of colors is a subjective process whereby the brain responds to the stimuli that are produced when incoming light reacts with the several types of cone cells in the eye. The spectral sensitivity function of the average human eye under daylight conditions (photopic vision) is defined by the CIE spectral luminous efficiency function V(λ) and it is valuable as a baseline for experimental purposes. By considering the influence of human eye sensitivity the *luminous transmittance* is defined as:


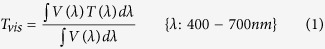


where *T*(*λ*) is corresponding to the wavelength depends transmittance spectra.

**Figure 13:**
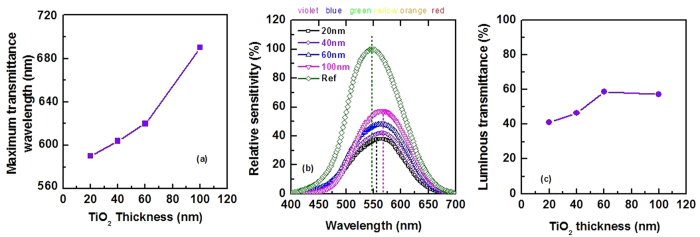
Impact of thickness of TiO_2_ layer on (**a**) maximum transmittance in visible wavelengths, (**b**) relative sensitivity and (**c**) luminous transmittance of TiO_2_/Cu/TiO_2_ THR.

As shown in Fig. 3, by tuning the thickness of TiO_2_ layer, peak position of transmittance can also be changed. To get deep inside about the impact of TiO_2_ layer thickness on the color of prepared TiO_2_/Cu/TiO_2_ transparent heat reflectors, a series of TiO_2_/Cu/TiO_2_ with different thickness of TiO_2_ are prepared. Thickness of Cu layer is fixed at 20 nm. The optical properties of TiO_2_/Cu/TiO_2_ transparent heat reflectors in the visible region are presented in Fig. 13. The peak position of transmittance spectra shifts toward higher wavelength as shown in Fig. 13(a). The shift of the visible transmittance peak indicates that the color of TiO_2_/Cu/TiO_2_ transparent heat reflectors depends on thickness of TiO_2_ layer.

Transmittance chromaticity and neutral appearing to human eye are the most crucial parameters in designing the heat mirrors for window applications. In Fig. 13(b), relative sensitivity of human eye is presented when the light passes through TiO_2_/Cu/TiO_2_ THR windows with different TiO_2_ layer thickness. The relative sensitivity of human eye under daylight condition is also shown in this figure. Using the windows with thicker TiO_2_ layer, causes the peak of the eye’s response slightly shifts toward the longer wavelength. As a result, eye’s responses slightly reduce for objects with violet color. The performance of TiO_2_/Cu/TiO_2_ THR with different thickness of TiO_2_ layer is evaluated in the visible wavelengths in terms of the luminous transmittance (In Fig. 13(c)). The luminous transmittance showed the highest value for a TiO_2_ layer thickness around 60 nm. Indeed at this specific thickness of TiO_2_, the color of TiO_2_/Cu/TiO_2_ THR appear more neutral to human eye. When TiO_2_ is thin, the metal layer dictates the color of appearance of the multilayer coating. For thicker TiO_2_, the color of the THR mainly attributed to the appearance of TiO_2_ color. The color of single TiO_2_ layer thin films depends on the thickness. For a given thickness of TiO_2_, certain wavelengths interfere constructively whilst others interfere destructively. The entire reflectance spectra shifts rightwards when TiO_2_ thickness increases, reflecting shorter wavelength of visible light (VIS) and IR while thinner TiO_2_ reflects longer wavelength of VIS and UV (Fig. 3). Thus the color of TiO_2_/Cu/TiO_2_ THR with thicker TiO_2_ appears bluish while thinner TiO_2_ appears reddish.

**Figure 14:**
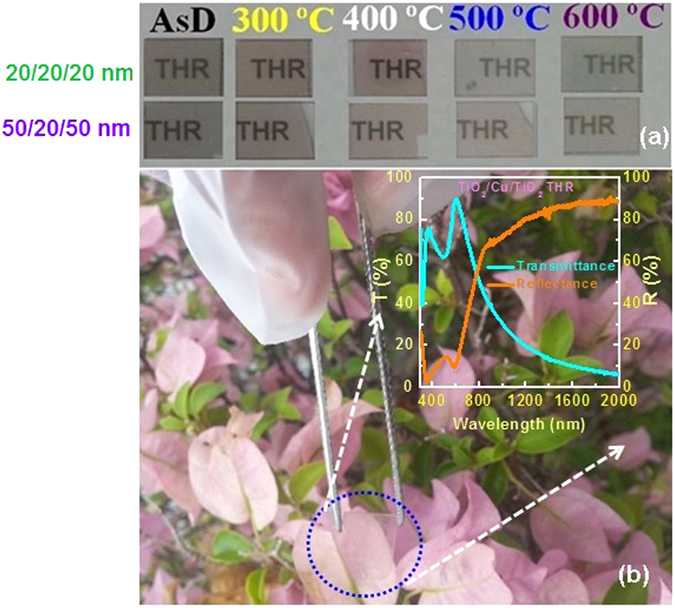
(**a**) Demonstration of TiO_2_/Cu/TiO_2_ heat reflector on glass substrate after thermal treatment. The total thickness of THR coating is ~60 nm (20 nm/20 nm/20 nm) and ~120 nm (50 nm/20 nm/50 nm). For the thicker layer of TiO_2_, more neutral color of the THR coating can be obtained. (**b**) Demonstration of the THR coating on glass (50 nm/20 nm/50 nm) for window application. The THR developed after thermal treatment at 500 °C for 1 min in nitrogen ambient. Inset of the figure (**b**) shows transmittance and reflectance spectra of the corresponding THR coating.

Figure 14 demonstrated the visible transmission of TiO_2_/Cu/TiO_2_ coating on glass substrate. Change in color with annealing temperature and TiO_2_ thickness can be seen in Fig. 14(a). For the thicker sample (total TiO_2_ thickness ~100 nm), more neutral colour was observed after annealing at 500 °C. Figure 14(b) demonstrates the performance of THR as window application after annealing at 500 °C for thicker TiO_2_. The slight decrease in transmittance after thermal annealing at 600 °C is due to the formation of interface oxide at Cu/TiO_2_. The metal-dielectric interface sharpness, which is resulted from inter-diffusion through TiO_2_ and Cu layer, is significantly influenced by annealing temperature. Indeed, due to out-diffusion oxygen from dielectric layer, the effective thickness of copper layer is reduced and consequently IR reflection is influenced. Thus, it is essential to keep the temperature below 600 °C to achieve high visible transmittance and IR reflectance with more neutral color.

According to Guo *et al*. solar absorber coating on a Cu substrate was stable upto 400 °C in air, and degradation of the coating occurred above 450 °C due to the diffusion of Cu^53^. The solar absorber coating based on Mo substrate was stable up to 450 °C in air and 800 °C in vacuum^54^. Moreover, thin film Mo between Cu and hafnium oxide (HfO_x_) layer could suppress the diffusion of Cu and thereby enhance the thermal stability^55^. Thus, the Cu diffusion and oxidation depend on the ambient of thermal treatment and also the Cu/metal oxide interface. The Cu metal out-diffusion occurred (in Cu nanowire structure) at 400 °C, when thermal treatment was done in air ambient^53^. In the present work, we have performed rapid thermal annealing in nitrogen ambient for short time (60 seconds) to improve the crystal quality of TiO_2_. Optical performance (visible transmittance and NIR reflectance) of the TiO_2_/Cu/TiO_2_ based THR significantly increases after thermal treatment up-to 500 °C. This also suggests that there is no Cu-out diffusion. It is worth to note that Braud *et al*. reported that there was no copper diffusion into 100 nm thick silicon oxide (SiO_2_) for temperature stress as high as 450 °C for one hour and for bias temperature stress (BTS) as high as 300 °C for 8 h at 1 MV/cm for Cu/SiO_2_ structure^56^. The Cu diffusion was not detected when the samples annealed under vacuum conditions. Furthermore, they also reported that the thin film of titanium (~5–20 nm) significantly reduced the Cu out-diffusion even at high temperature^56^. In our recent study on alloy oxides, presence of thin film TiO_2_ in Al_2_O_3_ significantly reduced the elemental out diffusion in gallium-arsenide based devices^57^. For the sputter grown TiO_2_/Cu/TiO_2_ structure, from SIMS analysis, it was found that there is a hump of Ti at TiO_2_/Cu interface, reveals formation of the Ti-rich oxide at the Cu/TiO_2_ interface. This observation is similar to the sputter grown CuO/Si interface (CuO was grown on silicon substrate using stoichiometry CuO target), where Cu-rich oxide interface layer was formed^49^,^50^. Thus, presence of thin Ti-rich oxide layer could also suppress the Cu-out diffusion into the oxide layer. Furthermore, XRD and HRTEM analysis showed crystal quality of the thin film TiO_2_ improved, whereas metal doped TiO_2_ is generally amorphous^58^,^59^. This also suggests that there was no out-diffusion of Cu into the TiO_2_. However, after thermal treatment at 600 °C, performance of the TiO_2_/Cu/TiO_2_ THR degrades, which is mainly due to the partial oxidation of Cu, as observed in SIMS and HRTEM analysis. Formation of thin layer CuO_x_ significantly changes the optical property of TiO_2_/Cu/TiO_2_ structure at high temperature, as CuO_x_ is a semiconductor with bandgap of 1.5 eV to 2.5 eV^51^,^60^,^61^.

In order to evaluate the adhesion of TiO_2_ on glass substrate, the scratch hardness of the coating is calculated with diamond indenter using ASTM G171-03 standard^62^. The samples were polished to support the scratch width (w) measurement. A constant z-directional load is applied by the diamond indenter and scratch mark has been occurred due to reciprocating motion of sliding of stylus. The optimal load was defined by performing the initial scratch experiment on the coating deposited on the substrate. The test was performed on Rtech, multifunctional tribometer to record the scratch width at the critical load. The average scratch width is calculated using optical microscope. The scratch hardness number (HSp) is calculated using equation


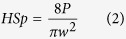


HSp is the scratch hardness number, P is the normal force, and w is the scratch width.

The results of scratch test are tabulated in Table 2. A critical load of 1 N was applied on coatings to compare results among all TiO_2_ coatings. All tests were performed at ambient temperature at following operating parameters; sliding distance −5 mm, sliding speed −0.01 m/s, sliding time −5 min. The scratches were performed perpendicular to the coatings surface. Adhesion between the glass substrate and the deposited coating observed good at the critical loading. The scratch hardness and wear resistance for thick TiO_2_ films were slightly higher, in contrast to thin TiO_2_ films. The higher scratch hardness of the thick TiO_2_ (Table 2) expedites the good adhesion of the coating deposited on glass substrate. The annealing temperature has positively influence on the scratch hardness of TiO_2_ films. With the increase in annealing temperature to 500 °C, the scratch hardness value increased significantly. For Cu-based THR, adhesive property between TiO_2_ and Cu is very strong. It is possible to coat THR on a single side of the glass. Henceforth, the sandwiched structure (glass/heat-reflecting-coating/glass) is not necessary to protect the THR from environmental effects. Cu-based THR is based on sputter deposition technique, which is industrial compatible. The Cu-based THR coating provides several advantages over silver based heat reflection coating; low cost raw materials and simple process technology. It can be deployed in large scale to the residential buildings.

## Conclusion

In summary, Cu-based transparent heat reflector (THR) coating has been demonstrated on glass substrate. TiO_2_/Cu/TiO_2_ multilayer thin film has been used for THR coating. Impact of TiO_2_ dielectric crystal quality on the performance of TiO_2_/Cu/TiO_2_ multilayer thin film was investigated in detail by XRD and HRTEM. It was shown that visible transmittance and IR reflectance can be enhanced simultaneously by improving the dielectric crystal quality through thermal treatment up to 500 °C. It was found that the heat reflector multilayer coating annealed at high temperature (~600 °C), oxygen can out diffuse into the metal layer and oxidized the thin Cu layer. Formation of thin interfacial layer between metal and dielectric was observed. Visible transmittance and IR reflectance degraded as a result of partial oxidation of thin Cu film. By tuning the thickness of TiO_2_ and annealing temperature, TiO_2_/Cu/TiO_2_ THRW with neutral color and high scratch hardness can be achieved. The present work shows promising characteristics of Cu-based THR and impact of thermal treatment to design the Cu-based low cost THRW for energy-saving building industry.

## Additional Information

**How to cite this article**: Dalapati, G. K. *et al*. Color tunable low cost transparent heat reflector using copper and titanium oxide for energy saving application. *Sci. Rep*. **6**, 20182; doi: 10.1038/srep20182 (2016).

## Figures and Tables

**Table 1:** Elemental compositions in TiO_2_/Cu/TiO_2_ heat reflector structure with total TiO_2_ thickness of 100 nm.

	Region 1			Region 2			Region 3			Region 4			Region 5		
	Cu (%)	Ti (%)	O (%)	Cu (%)	Ti (%)	O (%)	Cu (%)	Ti (%)	O (%)	Cu (%)	Ti (%)	O (%)	Cu (%)	Ti (%)	O (%)
AsD	3	53	44	3	57	40	92	4	4	3	54	33	3	55	32
500 °C	4	53	43	7	57	36	75	7	18	7	54	39	5	55	40
600 °C	5	51	44	8	56	36	63	9	28	8	54	38	6	55	39

**Table 2:** The results of scratch of TiO_2_/Cu/TiO_2_ heat reflector on glass substrate with total TiO_2_ thickness of 20 nm and 100 nm.

Thickness of TiO_2_ (nm)	Annealing temperature	HSp (GPa)
20	AsD	1.57
20	300 °C	1.63
20	500 °C	2.54
100	AsD	1.79
100	300 °C	1.63
100	500 °C	2.62
